# The Use of Revulsives in Diseases of the Nervous System

**Published:** 1875-11

**Authors:** Allan McLane Hamilton


THE USE OF REVULSIVES IN DISEASES OF THE NERVOUS SYSTEM.—*By Allan McLane Hamilton, M.D.*—For the production of the revulsive effect, we must bear in mind the important fact that application of the agent must be sudden and superficial. By observing these conditions we produce what is necessarily a shock.

Two forms of application which I consider of great value are, first, the actual cautery; second, the alternation of heat and cold. The use of the first in my hands during the past year has convinced me that for certain neuralgias no better remedy exists. Not only has it broken up attacks, but it has also most certainly brought about central changes resulting in healthy action of those parts. . . . .

Much depends upon the temperature of the iron. It is absolutely necessary that it should be brought to a white heat. A red-hot iron will produce deep action, and will destroy tissue by subsequent ulceration; the same results will follow the *slow passage* of the iron over the surface. The cautery-iron should invariably be white-hot; it should be drawn rapidly over the skin, and the result will be almost painless. The surface will be slightly discolored, and that is all, as the action is confined to the cuticle, which shrivels up and falls off in a few days. There has been a tendency of late to use the cautery-iron in convulsive affections, and it has been my privilege to see several patients who have gone through this ordeal without any benefit whatever, and have had their troubles aggravated by the nervous apprehension and dread associated with the white iron. As I have said before, the neuralgias are the neuroses that call for its use. . . . .

I have spoken of the uses of heat and cold for the relief of certain neuroses associated with trophic and circulatory defects. It remains for me to prove the efficacy of this treatment by the relation of experience. . . . .

Every neurologist is overrun by a class of patients that constitute a certain *approbrium medicorum.* These are patients with dysæsthesiæ, spinal irritation, and a few other maladies, confined chiefly to women, and dependent upon uterine diseases. After the employment of many remedies, and after finding hopeless results, constituted either by relapses or temporary improvement, the case is discharged. In these cases I found the alternate use of heat and cold to be followed by the most desirable effects, particularly in spinal irritation. Of twenty-six cases of this kind I have cured fifteen, and have greatly improved all the others. I had used phosphorus and electricity with indifferent success, but the alternate application of heat and cold to the spine produced a decided impression. I directed the patients to employ ice-bags, and hot flat-irons covered by flannel. In hysterical affections this mode of treatment was of great use, particularly those forms characterized by lividity of the surface and unconsciousness, with rigidity. . . . .

Dry heat and dry cold are much better as therapeutical agents than moist heat or moist cold. The effect is energetic ; the skin is influenced more quickly, as there is no fluid between to act as a non-conductor.

A consideration of the action of other revulsive agents would take many pages. I must say a word, however, of the virtues of the localized ether-spray to the spine. I can conceive of no better treatment for chorea and other convulsive disorders. I have witnessed its effects even in convulsive disease of adults. Shaking palsy, and the tremor of alcohol and sclerosis, may be moderated. It is safe to say that fifteen or twenty applications of the spray in most cases of chorea will effect a cure. I have tested it alone and with other remedies, and in several cases it cured the disease without adjuvant treatment.—*Philadelphia Medical Times.*

